# Detection and correction of incomplete duplicate 24-hour urine collections – theory and practical evidence

**DOI:** 10.11613/BM.2021.010706

**Published:** 2020-12-15

**Authors:** Raymond W Wulkan, Martin van der Horst

**Affiliations:** 1 Clinical Chemistry Laboratory, Maasstad Hospital, Rotterdam, The Netherlands; 2 Clinical Chemistry Laboratory, Treant Health Care Group, Scheper Hospital Location, Emmen, The Netherlands

**Keywords:** urine specimen collection, creatinine/urine, patient compliance

## Abstract

**Introduction:**

The intraindividual variability in urinary creatinine excretion is notoriously large. The aims of this study were to investigate the variability of duplicate consecutive 24-hour urinary creatinine excretions in patients and to develop a model for the detection and correction of discrepant creatinine excretions.

**Materials and methods:**

A group of 270 patients (82 men and 188 women) were included in the study. We collected the following data: urinary 24-hour volumes (volumetric/gravimetric) and urinary creatinine concentrations (Jaffé/enzymatic) on both collection days. We performed specific calculations to detect discrepant creatinine excretions.

**Results:**

In 60 patients (22%) discrepant collections were found. Among the remaining 78%, 22% of the patients collected very accurately (almost identical urinary creatinine excretions). In this subgroup the volume ratios and the creatinine concentration ratios behave inversely as in a dilution curve. A theoretical model and six collection scenarios were developed to detect, interpret and correct discrepant collections. Practical examples are given to illustrate the use of the model in successful correction of creatinine and other analytes for under- or overcollection.

**Conclusions:**

We conclude that missed or overcollected urine volumes are the largest source of variation in creatinine excretion. Discrepancies in consecutive duplicate 24-hour creatinine excretions can be detected and corrected with specific calculations by means of the presented model. The effectiveness of these corrections is demonstrated with examples from daily practice. These calculations can be easily automated.

## Introduction

The creatinine excretion in a 24-hour urine collection is a measure of muscular mass and renal function (*1*). This excretion may serve as an early indicator of sarcopenia (*2*, *3*). Accuracy of urine collection is therefore important, however the control of the completeness of collection is difficult. The methods to correct for incomplete collection, such as *p*-aminobenzoic acid excretion, the use of the creatinine index or the estimated creatinine excretion are far from ideal, as has been shown recently (*4*). In spite of the many reports of the high intraindividual variability of the 24-hour creatinine excretion, healthy, well instructed individuals may well collect with high precision, achieving a within person variation coefficient of 3.6% (*5*). The aims of this study were to investigate the variability of duplicate consecutive 24-hour urinary creatinine excretions in patients and to develop a model for the detection and correction of discrepant creatinine excretions.

Part of this work has been published previously in an observational study in Dutch language (*6*). The patient data, the development of detection limits and the findings from the literature presented here are the same as in the original publication. For clarity we have not omitted these parts, because this would disturb the line of reasoning too much. The new elements in the current article are the development of the mathematical model, the various scenarios derived from the model, the curved dependence of the creatinine ratio on the volume ratio, the detailed examples of aberrant collections and finally the equations that may be used for correction, as well as examples of corrections.

## Materials and methods

### Subjects

Adult outpatients of the Maasstad Hospital (Rotterdam, The Netherlands) and the Spijkenisse Medical Center (Spijkenisse, The Netherlands) collected duplicate, consecutive 24-hour urines for routine analysis (*i.e.* over 48 hours). Duplicate consecutive urine collections were analysed in order to eliminate the long term influences of changes in muscle mass, renal function or medication. The patient group consisted of 82 men (aged 21-85 years; median 61) and 188 women (aged 22-88 years; median 63) with hypertension, obesity or fractures. The urines were collected for the investigation of Cushing syndrome, pheochromocytoma, carcinoid tumor, porphyria, or osteoporosis/multiple myeloma in patients with fractures.

### Methods

Patients were instructed both verbally and in writing to empty their bladder early in the morning, to write down the time of day and to start the collection until the same time the next morning, to add the first portion of urine to the first collection and to write down again the time of day. When the collection time was deviating by less than one hour, the volume was corrected to the 1440 minutes of a complete day, otherwise collections were rejected. Urinary creatinine was measured by a Jaffé method (Dimension VISTA, Siemens Healthcare Diagnostics Inc., Tarrytown, USA) or by an enzymatic method (Cobas 6000, Roche Diagnostics GmbH, Mannheim, Germany). Both methods were calibrated to the assigned reference value of the external quality control material of the Dutch national external quality control organization. The urinary volume was either measured with a measuring cylinder or by weighing. The between day differences in creatinine excretion and volume were calculated by subtracting the values of the second day from those of the first day. The percentage difference in volume was determined by dividing the difference by the mean volume of both days. We chose this approach because it can not be known beforehand which of both collections is incorrect. All data were anonymised and were collected prospectively. The reference ranges for the excretion of creatinine are 8.0-22.0 mmol/24h for men and 6.0-17.0 mmol/24h for women. These reference ranges originate from the PREVEND study. This is a study with 2627 Dutch subjects living in the general population (*7*). The reference range for the 24-hour volume is 1000-3500 mL/24h (*8*).

### Statistical analysis

Standard statistical calculations were performed in Microsoft Excel 2010 (Microsoft, Redmond, USA). The curve fitting was done in GraphPad Prism, version 7.0a (GraphPad Software, San Diego, USA).

### Theoretical model

We describe a model for the situation of accurate collections in order to allow the systematical detection and correction of inaccurate collections. When both collections are correct, the creatinine excretions in 1440 min (mmol/24h) (Cr_24_) should be equal: Cr_24-1_ = Cr_24-2_ (where 1 and 2 represent the designation of the collection day) or V_1_ x c_1_ = V_2_ x c_2_ (where c represents the creatinine concentration, and V the urine volume in 1440 min (mL/24h)). This can be rewritten as (V_1_/V_2_) x (c_1_/c_2_) = 1. The mathematical product of V_1_/V_2_ and c_1_/c_2_ (R) will be abbreviated as R = 1; thus c_1_/c_2_ = 1/(V_1_/V_2_). When the volume ratio V_1_/V_2_ is plotted on the X-axis and the creatinine concentration ratio c_1_/c_2_ on the Y axis, the following hyperbolic curve is obtained: y = 1/x. In order to improve our understanding of the diverse presentations we used the model to describe six possible scenarios of incorrect collections.

#### The six scenarios

For every point of the line y = 1/x the R factor is equal to unity. This is the situation of perfect collections (R = (V_1_/V_2_) x (c_1_/c_2_) = 1). However, when R deviates from unity, the collections are less perfect. R can be larger or smaller than unity.

**Figure 1:**
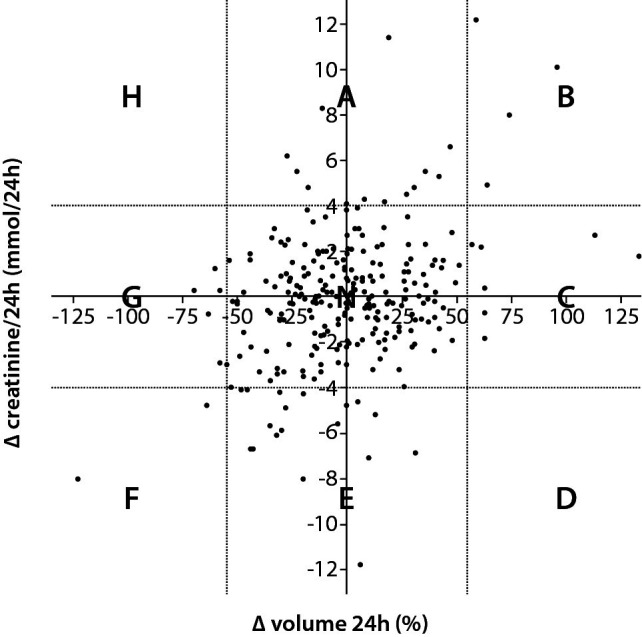
Scatter plot of the data of the patient group. The relative differences in 24-hour volume (X-axis) and the absolute differences in 24-hour creatinine excretion (Y-axis) are shown. The dotted detection limits divide the figure into nine sectors indicated in alphabetical order. These symbols are used in the discussion of the collection scenarios.

In case of R > 1, the creatinine excretion on the first day is larger than on the second day (V_1_c_1_ > V_2_c_2_). If we accept that c_1_ and c_2_ (and their ratio) are correct, there can only be three causes for R > 1: V_1_ is too large, V_2_ too small, or a combination of both (Figure 1).

1. The situation of an isolated too large V_1_ is rare. This occurs when the patient does not empty the bladder before collecting and adds this portion to V_1_. The patient is located in sector N or A dependent on the volume of the missed portion (5% of the patients are located in sector A) (Figure 1).
2. In case of an isolated grossly too low V_2_. The patient is located in sector B (1% of the patients) (Figure 1). In this situation the lowest volume (V_2_) may be seen in combination with the lowest creatinine (c_2_). This is an illogical combination, because volume and concentration behave inversely. The real V_2_ must have been higher and some volume has been missed. There might have been a spill of the second collection.
3. In situations of a too large V_1_ with a too small V_2_. The patient is located in sector A (Figure 1). The patient may not have emptied the bladder before collecting V_1_ and also mistakenly disposed the last portion of V_2_. Note that the volume difference doubles because both collections are affected. These double mistakes are less likely to occur.

In case R < 1, the creatinine excretion on the first day is smaller than on the second day (V_1_c_1_ < V_2_c_2_). If we accept that c_1_ and c_2_ (and their ratio) are correct, there can only be three causes for R < 1: V_1_ is too small, V_2_ too large or a combination of both (Figure 1).

1. An isolated grossly too low V_1_. It is rare (< 1% of the patients). In this situation the patient is located in sector F (Figure 1). In this situation the lowest volume (V_1_) may be seen in combination with almost equal creatinine concentrations. There might have been a spill of the first collection.
2. The situation of an isolated too large V_2_. It is rare (< 1% of the patients). The patient has extended the second collection beyond the second day. The patient is located in F (Figure 1).
3. In case of a too small V_1_ with a too large V_2_. The patient is located in sector E (Figure 1). This is the most frequently found situation (10% of the patients). It occurs when the patient mistakenly adds the last portion of the first day to the second collection on the morning of the second day. The displaced volume is roughly the content of a urine bladder (250-400 mL); this diminishes V_1_ but adds to V_2_ so the difference in volume is doubled.

The patient may be found in sector C or G (2% of the patients in either sector) when there is a large difference in water excretion, but only a small difference in creatinine excretion (Figure 1). The ratio V_1_/V_2_ is then large (in C) or small (in G) and therefore R may be larger or smaller than unity, respectively. The patient has collected correctly, but there is a large difference in water excretion. We remarkably did not find any of our patients in sector D or H. Both sectors represent illogical combinations of measurements. For instance, in sector D this would be V_1_>V_2_ in combination with c_1_>c_2_. When a patient is nevertheless found in sector D or H this may have been the result of a clerical error in the transcription of the volume.

## Results

The characteristics of the patient group are represented in Table 1. In short, they covered a broad range of age, volume and creatinine excretion.

**Table 1:** Demographic and measurement data of the patient group

	**N = 270**					
	**Male (N = 82)**			**Female (N = 188)**		
	**N**	**Mean (min-max)**	**SD**	**N**	**Mean (min-max)**	**SD**
Age (years)	79	61 (21-85)	14	178	63 (22-88)	15
Weight (kg)	38	94.5 (70-148)	17.5	79	78.3 (34-162)	21
Length (cm)	34	176.6 (110-192)	13.2	62	165.6 (147-188)	8.1
BMI (kg/m^2^)	32	29.8 (23.6-52.4)	6.1	63	29.4 (16.7-58.1)	8.3
Cr in plasma (µmol/L)	17	96 (60-143)	26.6	52	68 (46-124)	13.3
Cr in urine (mmol/L)	82	9.4 (3.0-20.2)	4.1	188	5.8 (1.5-14.2)	2.7
Cr_24_ (mmol/24u)	82	14.4 (6.2-26.8)	4.2	188	8.6 (1.8-16.1)	2.9
Volume (mL)	82	1767 (514-3888)	651	188	1737 (468-4090)	673
Age is presented as median (range). Cr - creatinine concentration. Cr_24_ - 24-hour creatinine excretion. SD – standard deviation. BMI - body mass index. For some patients not all data were available.						

### Choice of detection limits

Twenty-three of the 270 patients had a large (arbitrarily > 5.0 mmol/24h) absolute difference between both creatinine excretions and were removed as outliers, leaving 247 patients. From this population a standard deviation in creatinine excretions of 2.0 mmol/24h was calculated, and a 95% confidence interval of ± 4.0 mmol/24h. We used this interval as a definition of acceptable collections, which also means that values outside this interval are considered as having an unacceptable difference between both collections. We adopted limits from the difference of 24-hour volume as ± 55% from the literature (*9*). In our group of patients 60/270 (22%) were found outside the detection limits, indicating unacceptable collection errors.

### Experimental evidence of the theoretical model

**Figure 2:**
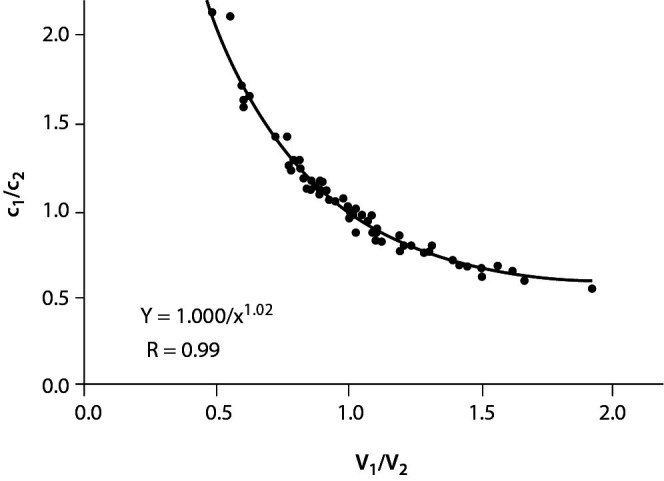
Volume ratio against the creatinine concentration ratio plotted for the 60 patients who collected accurately.

We selected from our group of 270 patients those with an absolute value of the creatinine excretion difference in the first and second day (|∆Cr_24_|) < 0.5 mmol/24h (near to perfect collections). Sixty patients (22%) fulfilled this criterion. From this subgroup we plotted the volume ratio (V_1_/V_2_) against the concentration ratio (c_1_/c_2_) and obtained the line y = 1.0/x^1.02^ with a correlation coefficient of 0.990 (Figure 2).

This line is virtually identical to the line predicted from the theoretical model, representing accurate collections, for which the R factor is close to unity. The prediction line may be described by the situation of a beaker containing one liter of urine to which one litre of water is added: the volume doubles and the solute concentration halves. The line describes a common dilution situation, where the ratio of c_1_ and c_2_ is determined exclusively by the addition of water. We were greatly surprised to find this experimental evidence of such a simple situation in a complex biological environment like the kidney. This finding has several important implications. First, it is proof that a substantial number of patients are able to collect to near perfection. Second, the line permits the correction of faulty volumes to the volumes of a correct collection, provided that the ratio c_1_/c_2_ is correctly measured and remains constant over both collection days (for the calculations of volume corrections see Appendix 1).

### Case descriptions and corrections

To illustrate the use of the model we have selected seven cases from daily routine (Table 2 and Figure 1).

**Table 2:** Examples of patient cases with incorrect collections

**Case**	**1**	**2**	**3**	**4**	**5**	**6**	**7**
Age (y)	60	62	53	68	55	66	67
Gender	F	M	M	F	F	F	F
V_1_ (mL)	772	2232	1980	1860	1874	1800	422
V_2_ (mL)	1232	2806	693	890	2392	3500	1400
Cr_1_ (mmol/L)	13.5	8.2	8.4	4.6	4.3	2.8	14.3
Cr_2_ (mmol/L)	7.6	4.2	9.6	9.5	6.4	2.8	4.4
Cr_24-1_ (mmol/24h)	10.4	18.3	16.6	8.6	8.1	5.0	6.0
Cr_24-2_ (mmol/24h)	9.4	11.8	6.7	8.5	15.3	9.8	6.2
ΔCr_24_ (mmol/24h)	1.0	6.5	9.9	0.1	- 7.2	- 4.8	- 0.2
ΔV%	- 46%	- 23%	96%	71%	- 24%	- 64%	- 107%
R	1.11	1.55	2.48	1.01	0.53	0.51	0.97
Sector	N	A	B	C	E	F	G
Suffix 1,2 - designation of the collection day. V - 24-hour volume. Cr - creatinine concentration. Cr_24_ - 24-hour creatinine excretion. ∆Cr_24_ - difference between consecutive creatinine excretions. ∆V% - relative difference between consecutive 24-hour urine volumes. R - the ratio of Cr_24-1_/Cr_24-2_. Sector - position in the sectors as designated in Figure 1.							

Case 1. The ∆Cr_24_ is amply below 4.0 mmol/day so the patient has collected correctly. The relative difference between consecutive 24-hour urine volumes (∆V%) is - 46%, which is just normal (above - 55%). The R factor is normal. The patient is found in sector N. Volume correction is not necessary.

Case 2. The patient has an increased ∆Cr_24_ and a normal ∆V% and is found in sector A. The patient has overcollected on the first day. The initial 24-hour calcium excretions were 2.8 and 1.9 mmol/24h and were recalculated after volume correction to be 1.8 and 1.9 mmol/24h respectively.

Case 3. In spite of the large difference between V_1_ and V_2_, the creatinine concentrations are almost equal, which is illogical. The ∆Cr_24_ is strongly increased. The patient has strongly undercollected on the second day, and is found in sector B. The initial 24-hour normetanephrine excretions were 21.2 and 8.1 mmol/24h and were recalculated after correction of V_2_ to be 21.2 and 20.5 mmol/24h. The R factor is strongly increased.

Case 4. In spite of the large difference between V_1_ and V_2_, the patient has collected meticulously in view of the very low ∆Cr_24_ and the R factor which is almost equal to unity. The ∆V% is 71%, which is above normal. The patient is found in sector C and there is a slightly increased difference in water excretion. Volume correction is not necessary. Despite comparable urine volumes this patient contrast sharply with Case 3, where there is a gross collection error.

Case 5. The highest creatinine concentration is found in combination with the highest volume on day two, which is an illogical combination. The ∆Cr_24_ is strongly decreased, as is the R factor. The patient is found in sector E. There has been an undercollection on the first day and an overcollection on the second day. The initial 24-hour calcium excretions were 7.1 and 13.3 mmol/24h and were recalculated after volume corrections to be 9.6 and 9.5 mmol/24h respectively.

Case 6. Despite a large difference in volume between V_1_ and V_2_, the creatinine concentrations are identical, which is illogical. The ∆Cr_24_, the ∆V% and the R factor are all decreased. The patient is found in sector F. The patient has grossly overcollected on the second day. The initial 24-hour metanephrine excretions were 0.25 and 0.52 mmol/24h and were recalculated after volume correction to be 0.25 and 0.27 mmol/24h respectively. Likewise, the 24-hour sodium excretions were 58 and 126 mmol/24h and were corrected to 58 and 64 mmol/24h respectively.

Case 7. Despite a large difference in volume between V_1_ and V_2_, in view of the very low ∆Cr_24_ and the R factor close to unity, the patient has collected meticulously. The ∆V% is largely decreased and the patient is found in sector G, indicating a large difference in the excretion of water. Volume correction is not necessary.

The cases presented above demonstrate the effectiveness of the model in solving discrepancies in the excretion of calcium, metanephrines, or cortisol (not shown). These corrections also demonstrate that there is only negligible influence from other interfering factors. We have implemented this authorisation in our daily routine practice. We have implemented an alert for incorrect collections in our laboratory information system. The clinical biochemist then decides to make a comment for the clinician and/or recalculate the results. We have also automated the correction calculations in an Excel program, which presents the classification sector, the R factor, the corrected volumes according to the different scenarios and optionally, the corrected 24-hour excretions of other solutes.

## Discussion

As mentioned in the section Results we calculated the detection limits in patients as ± 4.0 mmol/24h for the difference in creatinine excretion. These limits are similar to the early literature where critical differences in creatinine excretion were reported: 3.5 mmol/24h for women and 4.3 mmol/24h for men. However, these were calculated from a group of only 15 healthy individuals (*10*). More recently, in an elaborate study an optimal critical difference in creatinine excretion was defined as ± 40% (*11*). When we apply this percentage to the mean creatinine excretion in our patient group (10.4 mmol/24h) we obtain the same detection limits of ± 4.0 mmol/24h for the difference in creatinine excretion. Limits for the difference in 24-hour volume have been reported previously (*9*). These authors calculated a critical difference of 54.5% from a group of 459 healthy individuals. We adopted these limits as ± 55%.

We studied the literature for the magnitude of factors influencing the creatinine excretion.

The effect of boiled meat on the urinary creatinine concentration has been documented: 0.7 mmol/24h increase/100g of boiled meat, and 1.5 mmol/24h/100g increase when compared to vegetarians (*12*, *13*). These increases are too small to cause detection as an inappropriate collection by our method. The effect of creatine ingestion on the urinary creatinine excretion requires repeated ingestion of large doses of creatine (20 g/day): in such a regimen the urine creatinine excretion peaks at day five (to 5 mmol/24h increase) and then stabilises at a lower increase of 2.8 mmol/24h (*14*). Menstruation has been shown to have no influence on the creatinine excretion (*15*). The effect of strenuous exercise has been well documented: 30-50 km of walking during eight hours induced a mean increase of 3.5 mmol/L in creatinine excretion (*16*). Cycle ergometry until exhaustion caused a mean increase of 2.3 mmol/24h, range 0.2-4.2 mmol/24h (*17*). When the exercise was moderate, others found only nonsignificant changes (*18*). Taken together it is highly unlikely that the factors mentioned above will lead to a false signal of urine collections as being inappropriate in our group of patients.

In some studies of urine collections, data of self-reported missed volumes have been published: a mean missed volume of 322 mL or 280 mL with a wide range of 20-735 mL (*19*, *20*). Likewise, when the literature is searched for reports of the urinary bladder volume, wide ranges are found: 120-465 mL, 15-750 mL (mean 322 mL for men, 255 mL for women) and 192-349 mL (25th-75th percentile, mean 246 mL in a large study of 1449 men) (*21*-*23*). It is conceivable that a patient mistakenly under- or overcollects by one or even by two bladder volumes. If we assume a mean bladder volume of 300 mL and combine this with a mean patient creatinine concentration of 10 mmol/L, the difference in excretion between two days of a missed portion is 3 mmol/24h or even 6 mmol/24h. It is obvious that the effect of under- or overcollection by far outweighs the effect of the other factors. When we realised this we decided to develop a model based on the variation in creatinine excretion caused by under- or overcollection alone. Although we realise that this is an idealised situation, experience with the model in daily routine taught us that it proves very helpful to solve discrepancies in the interpretation of differences in excreted solutes.

There are a number of limitations in this study. The model rests on the assumption that under- or overcollection is dominant in the variability of the creatinine excretion. This may not always be the case with young healthy individuals instead of middle aged or elderly patients. The model will not detect under- or overcollection when it occurs in the same manner on both days. It also relies on accurately measured urine volumes and creatinine concentrations. In our hospital most of the collections are done consecutively. Therefore we chose to study this paired collections. Due to this practise in our hospital we had no data to study whether the suggested delta check can be applied between two 24-hour urine collections several weeks apart. This would be interesting, although it would open the door to long term influences on the creatinine excretion, such as illness or medication changes.

The model must be used with caution in dialysis patients because these patients with advanced renal failure have artificial pathways of creatinine removal. We did not study the use of the model in paediatric populations.

Lastly, in one location of the hospital the urine weight is measured in grams, in the other location the volume is measured to the nearest 10 mL. With an average excretion of 1000 mL the maximal error is 1% and can be neglected comparative to the large errors that we discuss.

There are also some strong points in this study. This is the first study with a standardised authorisation of duplicate 24-hour urine collections, which may be easily automated in the laboratory information system. It is one of the largest studies in the literature of duplicate urine collections. The model offers an approach to understand the effects of missed volumes on the excretion of other urine solutes and allows to correct for these effects. This minimises discrepancies and sharpens the interpretation. We also have presented evidence that patients are well able to collect to near perfection and that in this situation the urinary creatinine concentration is solely dependent on the amount of water excreted, which may vary up to a factor two between consecutive days.

The identification of volume errors as the major source of variation raises the question whether instructions for urine collection are always given and followed in a precise manner. This could be the subject of future research. We have shown that 22% of the patients is able to collect very accurately, that 56% of the patients collect with moderate precision and that 22% of the patients collect with highly unacceptable precision. The reader should realise that a critical difference of ± 4.0 mmol/24h amounts to an approximate 40% in relative terms. In contrast, one fifth of our patients were able to collect within ± 0.5 mmol/24h (approximately 5%). Currently we do not know why these patients are able to collect to near perfection. Taken together these findings suggest that there is ample room for improvement. Apart from failing instructions, there could be other reasons for inadequate collections such as physical or psychiatrical limitations. This too could be the subject of further research.

In conclusion, the variability in creatinine excretion in our patient group is comparable to that of earlier studies (results not shown). Study of the literature identifies under- or overcollection as the major source of variability in creatinine excretion. We have developed a model for the authorisation of duplicate, consecutive 24-hour urine collections. This model is used in our hospital to detect, to interpret and to correct discrepant collections. We have demonstrated the effectivity of the model with examples from the daily practice. We have also provided the calculations in order to facilitate the reader in automation of the model. The results of this study may also be of use to laboratories that express the excretion of certain analytes as a ratio relative to creatinine, an approach that has been criticized (*24*, *25*). We hope that our work will contribute to a revival of interest in the subject and will lead to further improvements in the accuracy of 24-hour urine collection.

## Appendix 1 Calculation of the volume corrections

The mathematically corrected 24-hour volumes V_1c_ and V_2c_ must lie on the curve y = 1/x.

### Correction of V_1_ only (scenarios 1 and 4)

c_1_/c_2_ = 1/(V_1c_/V_2_) or V_1c_/V_2_ = c_2_/c_1_.

When V_1_ is too large, the correction volume z has to be subtracted:

V_1c_ = V_1_ – z;

(V_1_ – z)/V_2_ = c_2_/c_1._

From this follows:

z = V_1_ – (V_2_ x c_2_/c_1_) and

V_1c_ = V_2_ x c_2_/c_1_.

### Correction of V_2_ only (scenarios 2 and 5)

c_1_/c_2_ = 1/(V_1_/V_2c_) or V_1_/V_2c_ = c_2_/c_1_.

When V_2_ is too small, z has to be added:

V_2c_ = V_2_ + z;

V_1_/(V_2_ + z)/ = c_2_/c_1._

From this follows:

z = (V_1_ x c_1_/c_2_) – V_2_ and

V_2c_ = V_1_ x c_1_/c_2_.

### Correction of both V_1_ and V_2_ (scenarios 3 and 6)

c_1_/c_2_ = 1/(V_1c_/V_2c_) or V_1c_/V_2c_ = c_2_/c_1_.

When V_1_ is too small and V_2_ is too large, both volumes have to be corrected:

(V_1_ + z) / (V_2_ – z) = c_2_/c_1_

(V_1_ x c_1_) + (z x c_1_) = (V_2_ x c_2_) - (z x c_2_)

z (c_1_ + c_2_) = (V_2_ x c_2_) - (V_1_ x c_1_)

z = [(V_2_ x c_2_) - (V_1_ x c_1_)] / (c_1_ + c_2_).

In the inverse situation, the correction volume z changes sign. When V_1_ is too large and V_2_ is too small, both volumes have to be corrected:

(V_1_ - z) / (V_2_ + z) = c_2_/c_1_

(V_1_ x c_1_) - (z x c_1_) = (V_2_ x c_2_) + (z x c_2_)

z (c_1_ + c_2_) = (V_1_ x c_1_) - (V_2_ x c_2_)

z = [(V_1_ x c_1_) - (V_2_ x c_2_)] / (c_1_ + c_2_) = - [(V_2_ x c_2_) - (V_1_ x c_1_)] / (c_1_ + c_2_).
