# Erratum to: Detection and correction of incomplete duplicate 24-hour urine collections – theory and practical evidence

**DOI:** 10.11613/BM.2021.011201

**Published:** 2021-02-15

**Authors:** Raymond W Wulkan, Martin van der Horst

**Affiliations:** 1 Clinical Chemistry Laboratory, Maasstad Hospital, Rotterdam, The Netherlands; 2 Clinical Chemistry Laboratory, Treant Care Group, Scheper Hospital Location, Emmen, The Netherlands

This is a correction of Biochem Med (Zagreb) 2021;31(1):010706, DOI: https://doi.org/10.11613/BM.2021.010706 .

Since the publication of the article, the authors have noticed some errors: on page 5, right column, under the heading Case 3, the concentrations of normetanephrine should be in µmol/24h instead of mmol/24h; and on page 6, left column, under the heading of Case 6, the concentrations of metaphrine should be in µmol/24h instead of mmol/24h. We apologize to the authors for any inconvenience caused to the readers.

